# Supplementary material for: PrEP knowledge and perceptions among women living in North Carolina public housing communities
Source: PLoS One. 2025 Mar 31;20(3):e0320093. doi: 10.1371/journal.pone.0320093 (PMC11957289; doi:10.1371/journal.pone.0320093)
Supplement: Attachment — Submitted filename: IFE PLOS RR_Response to Reviewers rev.docx [file pone.0320093.s002.docx]

**Response to Reviewers**

We thank the reviewers for their thoughtful comments on our manuscript. We have revised the manuscript in response to these comments as summarized below.

**Reviewer #1**

**1. (Introduction) Social and economic structures as barriers to PrEP is discussed but there is no mention of structural racism called out.**

**Response:** We have added a statement addressing the role of structural racism in shaping social determinants of health and the health outcomes (beginning line 40 of the Introduction).

**2. (Discussion) More details could be added to the discussion with regard to: a) the implications of the results for new forms of PrEP; b) the influence of policy for the study population primarily made up of Medicaid recipients; c) the influence of men on PrEP education and uptake on women in this population.**

**Response:** We have addressed each suggestion as follows: a) We have added a statement regarding the implications of our results for long-acting PrEP methods (line 469). The preceding part of the same paragraph also addresses our direct results relating to long-acting PrEP; b) We have added discussion of the potential impact Medicaid policy in the study setting (beginning on line 425); c) Our results do not directly address the role of men in women’s PrEP education, thus we have chosen not to include this in the Discussion.

**3. Re-consider the use of language around "risk."**

**Response:** We have replaced this term throughout the manuscript and provide examples of the revisions below, following the guidance from ViiV cited by the reviewer (<https://viivhealthcare.com/content/dam/cf-viiv/viivhealthcare/en_US/pdf/from-risk-to-reasons-reframing-hiv-prevention-and-care-for-black-women-spreads.pdf>) and NIAID (<https://www.niaid.nih.gov/sites/default/files/niaid-hiv-language-guide.pdf>) wherever possible.

| **Old term** | **New term** |
| --- | --- |
| HIV risk factors | HIV-associated factors |
| Risk perception | perceived HIV acquisition/exposure chances |
| Women with HIV risk indications | Women with reasons for HIV prevention |

**4. There is an opportunity to mention in the discussion a future need for research to shift to addressing and examining social determinants of health in a way that interventions and programs will reflect strategies that respond to these SDOH.**

**Response:** We have added statements to the Discussion (beginning line 486) highlighting the importance of addressing not only knowledge and attitudinal factors but also social determinants of PrEP access. We further provide context by sharing that the results of this study were used to develop an awareness-raising campaign that complemented a mobile PrEP clinic program, aimed at addressing the social determinant of health care access.

**Reviewer #2**

**1. (Abstract) The abstract could benefit from further interpretation of key statistics presented.**

**Response:** We have clarified the significance of key results presented in the abstract, including an interpretation of the results regarding the fact that few women had previous knowledge of PrEP but despite this many were interested in it and expected positive community attitudes toward PrEP.

**2. (Abstract) The final sentence could offer a stronger closing message about the implications of these results for future PrEP outreach and intervention strategies.**

**Response:** We have strengthened the concluding remarks in the abstract to offer more specific recommendations for future PrEP outreach, including the need to specifically address cisgender women, provide transparent information on side effects, and offer destigmatized messaging about why women may consider PrEP use.

**3. (Abstract) Add framing of the problem addressed by this paper in a broader context of PrEP uptake in underserved populations to emphasize the importance of the findings.**

**Response:** Due to word limit constraints, we were unable to add this broader context in the abstract but address this in the Introduction, where we discuss PrEP uptake among cisgender women in the national context.

**4. (Introduction) It would be helpful to more clearly highlight gaps in the existing literature, especially in PrEP campaigns targeted at women in low-income communities.**

**Response:** While there have been online campaigns targeting cisgender women in the US, to our knowledge no evidence-based approaches for community-level communication targeting cisgender women are currently available, but one is in development (line 60).

**5. (Introduction) The introduction needs more transition sentences linking various sections.**

**Response:** We have reviewed the Introduction and added transitional phrases to improve flow and clarity.

**6. (Introduction) Address specific PrEP uptake barriers such as those in the findings in the introduction to provide context for the results.**

**Response:** We have added a discussion of PrEP uptake barriers related to side effects, cost, and stigma to the Introduction (line 45).

**7. (Methods)The sampling is described as convenience-based. A more detailed explanation of why this method was chosen, along with its potential limitations should be provided.**

**Response:** We have added our rationale for convenience-based sampling in the Methods section (line 85). In the Limitations section, we discuss the potential biases associated with the sampling approach. Convenience sampling was selected due to unavailability of a resident list or contact information for probabilistic sampling. Survey participants were recruited on a convenience basis, thus results may not represent the experiences and opinions of all women living in the included public housing communities. To encourage representation (albeit not through probabilistic methods), we conducted outreach to most public housing communities in the sampling frame on repeated occasions and varied our recruitment locations, times, and channels within each community to recruit as broadly as possible.

**8. (Methods) Address any potential limitations with respect to non-response or how different sociodemographic factors were controlled for in the analysis.**

**Response:** We have added a statement in the Limitation sections regarding survey non-response (beginning line 502). Because our analyses did not include causal or multivariate models, no controls were included. Rather, we present descriptive statistics and bivariate comparisons to offer descriptive understanding of PrEP perceptions among women with differing reasons for HIV prevention. We have added this caveat to the Limitations (beginning line 507).

**9. (Results) The tables are dense and could be simplified or split into more digestible sections. Key findings from the tables could also be highlighted or summarized in the text.**

**Response:** To make the tables less dense, we made the following changes. In Table 1 and Table 7, we collapsed some of the categories reported in the descriptive statistics for digestibility. We split Table 2 into two separate tables (distinguishing between results regarding HIV-associated factors and personal HIV perceptions). Key findings from the tables are also summarized in the text.

**10. (Results) More quotes would help bring the participant perspectives from the focus groups to life.**

**Response:** We have ensured that each major finding from the focus groups is supported by at least one participant quote.

**11. (Results) The manuscript mentions differences between women with HIV-associated factors and those without but could delve deeper into explaining these differences (e.g., using subgroup analyses).**

**Response:** These analyses are outside the scope of the current paper in which our goal is to characterize a broad range of constructs related to PrEP interest and perceptions in the study population. We are exploring the feasibility of future analyses to better understand subgroup differences for a future publication.

**12. (Discussion) Augment the discussion of PrEP uptake in similar populations to contextualize study findings.**

Response: There is limited information on PrEP uptake among similar populations, but we have added available statistics to provide context to the Discussion (line 419).

**13. (Discussion) Since side effects and costs are recurring concerns, more emphasis should be placed on how future campaigns can specifically address these barriers.**

**Response:** We have added recommendations to address concerns around side effects as well as the cost of PrEP and associated care to the Discussion (beginning line 422). These recommendations include transparency in side effect information and clarification on the commonality of each side effect, and information on diverse PrEP access points which may have different implications for cost (e.g. public vs. private providers, local vs. telehealth options).

**14 (Discussion) Details should be added to the Limitations regarding the generalizability of the findings to women outside public housing.**

**Response**: We have added a statement to the Limitations regarding the potential inability to generalize the findings to other populations and settings in the US and globally and offered suggestions for the potential relevance of our results to similar populations and communities in the Southeastern US (line 511).

**15. (Conclusions) Add more actionable takeaways or specific next steps that could guide future researchers, practitioners, or policymakers in PrEP intervention design.**

**Response**: We have added specific recommendations to the Conclusions highlighting features critical to the success of PrEP education campaigns based on our results, such as delivery by trusted sources and an emphasis on choice. We also outlined actionable recommendations for future interventions to promote PrEP awareness and access among cisgender women.

**16. (Citations) Ensure the that the most recent relevant studies on PrEP uptake among women are included.**

**Response**: We have updated the literature review and added citations for relevant studies published in the past three years.

**17. (Citations) Double-check for citation consistency, especially in terms of formatting. All references should be thoroughly proofed to follow journal guidelines.**

**Response**: We have edited the in-text citations and references cited to ensure formatting consistency and compliance with journal guidelines.

**Additional changes made in response to Journal Requirements:**

We have addressed journal requirements as follows:

- We have reviewed journal style guidelines and have ensured that our title page, manuscript, and references meet PLOS ONE's style requirements.
- We have updated our data statement to indicate an institutional, non-author point of contact for data access.
- We have reviewed our reference list to ensure that it is complete and correct. Specifically, we have added references to bring our literature review up to date and to address new points in the Introduction and Discussion (per responses to reviewers above). We have also corrected in-text citation and reference formats to meet journal formatting standards.
- We have amended the funder role statement in the financial disclosure as follows (edited text underlined):
  - *This research was funded by Gilead Sciences. Additional investigator and administrative support were provided by the National Institute of Mental Health (K01 MH121186) and the National Institute of Allergy and Infectious Disease (P30 AI050410). The funders had no role in study design, data collection and analysis, decision to publish, or preparation of the manuscript.*
